# Potential effects of omega-3 fatty acids on anemia and inflammatory markers in maintenance hemodialysis patients

**DOI:** 10.1186/2008-2231-22-11

**Published:** 2014-01-07

**Authors:** Afshin Gharekhani, Mohammad-Reza Khatami, Simin Dashti-Khavidaki, Effat Razeghi, Alireza Abdollahi, Seyed-Saeed Hashemi-Nazari, Mohammad-Ali Mansournia

**Affiliations:** 1 Resident of Clinical Pharmacy, Faculty of Pharmacy, Tehran University of Medical Sciences, Tehran 1417614411, Iran; 2 Nephrology Research Center, Tehran University of Medical Sciences, Tehran 1417614411, Iran; 3 Faculty of Pharmacy, Tehran University of Medical Sciences, Tehran 1417614411, Iran; 4 Vali-e-Asr Hospital, Tehran University of Medical Sciences, Tehran 1417614411, Iran; 5 Department of Epidemiology, School of Public Health, Shahid Beheshti University of Medical Sciences, Tehran 1417614411, Iran; 6 Department of Epidemiology and Biostatistics, School of Public Health, Tehran University of Medical Sciences, Tehran 1417614411, Iran

**Keywords:** Anemia, Hemodialysis, Inflammation, Omega-3 fatty acids

## Abstract

**Background:**

Anemia is a common complication among hemodialysis (HD) patients. Although intravenous iron and erythropoiesis-stimulating agents revolutionized anemia treatment, about 10% of HD patients show suboptimal response to these agents. Systemic inflammation and increased serum hepcidin level may contribute to this hyporesponsiveness. Considering the anti-inflammatory properties of omega-3 fatty acids, this study aimed to evaluate potential role of these fatty acids in improving anemia and inflammation of chronic HD patients.

**Methods:**

In this randomized, placebo-controlled trial, 54 adult patients with HD duration of at least 3 months were randomized to ingest 1800 mg of either omega-3 fatty acids or matching placebo per day for 4 months. Anemia parameters including blood hemoglobin, serum iron, transferrin saturation (TSAT), erythropoietin resistance index, and required dose of intravenous iron and erythropoietin, and serum concentrations of inflammatory/anti-inflammatory markers including interleukin (IL)-6, tumor necrosis factor (TNF)-α, IL-10, C-reactive protein (CRP), hepcidin, ferritin, intact parathyroid hormone (iPTH), and ratios of IL-10 to IL-6 and IL-10 to TNF-α were measured at baseline and after 4 months of the intervention.

**Results:**

45 subjects (25 in the omega-3 and 20 in the placebo group) completed the study. No significant changes were observed in blood hemoglobin, serum iron, TSAT, and required dose of intravenous iron in either within or between group comparisons. Additionally, erythropoietin resistance index as well as required dose of intravenous erythropoietin showed no significant change in the omega-3 group compared to the placebo group. Although a relative alleviation in inflammatory state appeared in the omega-3 group, the mean differences of inflammatory and anti-inflammatory markers between the two groups did not reach statistically significant level except for IL-10-to-IL-6 ratio and serum ferritin level which showed significant changes in favor of omega-3 treatment (P <0.001 and P = 0.003, respectively).

**Conclusion:**

Omega-3 fatty acids relatively improved systemic inflammation of chronic HD patients without any prominent benefits on anemia. However, future well-designed studies on larger number of patients may determine utility of omega-3 fatty acids in HD patients with respect to inflammation and anemia.

## Background

Anemia occurs as one of the most common complications of end-stage renal disease (ESRD) in patients undergoing HD [1,2]. It is manifested typically as normocytic and normochromic anemia with normal cellularity of bone marrow [3]. Although erythropoietin (EPO) deficiency is the major reason in the pathogenesis of anemia in ESRD patients, other factors including shortened erythrocyte survival, blood loss, iron and other nutritional deficiencies, hemolysis, oxidative stress, and presence of uremic inhibitors of erythropoiesis seem to play also important roles [4]. Most patients with ESRD and anemia exhibit adequate response to erythropoiesis-stimulating agents (ESA); however, about 10% of the subjects remain hypo- or non-responsive to ESA [2]. Adequate iron supply is necessary to obtain optimal benefit from the ESA therapy. Considering the accelerated erythropoiesis with the initiation of ESA therapy combined with the ongoing uremia- and dialysis-related iron losses, HD patients on ESA are highly predisposed to development of iron-restricted erythropoiesis since the rate of iron release from stores and its delivery to erythroid marrow fail to supply the increased marrow demand. Intravenous (IV) iron supplementation can properly correct limited availability of iron to erythroid marrow and improve hemoglobin concentration [1,5].

On the other hand, dialysis patients have a chronic inflammatory state which may contribute to iron-restricted erythropoiesis via decreasing the mobilization of iron from the reticulo-endothelial system to circulating transferrin, a situation which is associated with the reduced response to ESA therapy and IV iron supplementation [1,3,6]. Several underlying factors have been noted to be responsible for chronic systemic inflammation in HD patients, including the uremic milieu, enhanced infection episodes, greater generation of pro-inflammatory cytokines, widespread atherosclerosis, and the use of bio-incompatible dialysis membrane [3]. Recently, there has been an increasing attention to inflammation in HD patients as a possible cause of disordered iron homeostasis as well as anemia refractory to ESA treatment. Discovery of hepcidin, a 25-amino acid peptide hormone mainly produced in the liver, has dramatically increased our understanding of the molecular basis of iron metabolism over the several past years. It is regarded as the key regulator of iron entry into the systemic circulation [7], and is up-regulated by increased iron stores and inflammation and down-regulated by hypoxia, anemia, and iron deficiency [3,8]. Hepcidin acts by binding to cellular iron exporter ferroportin which is present on the cell membrane of the enterocytes, macrophages and hepatocytes and leads to its internalization and degradation [9]. Thus, the net effect of increased hepcidin synthesis is the reduced absorption of dietary iron and sequestration of iron in hepatocytes and macrophages. It has been reported that dialysis patients have higher serum levels of hepcidin [10-14], probably due to the impaired renal excretion coupled with an enhanced generation secondary to inflammation and iron excess [15]. In addition to limiting iron availability for erythropoiesis, hepcidin may also contribute directly to ESA resistance by an inhibitory impact on erythroid progenitor proliferation and survival [16].

Omega-3 fatty acids have been shown to provide dialysis patients with a host of biochemical and clinical benefits [17]. Nevertheless, it has been shown that dietary fish consumption, as a main source of omega-3 fatty acids, in chronic HD patients was much less than that recommended by American Heart Association, resulting in suboptimal blood levels of omega-3 fatty acids [18]. Similarly, significantly lower concentration of omega-3 fatty acids in erythrocyte membrane phospholipids has been found in HD patients compared with the healthy controls [19], largely due to less consumption of fish or fish-derived products in this patient population [17]. However, the findings of previous studies on anti-inflammatory effects of omega-3 fatty acids supplement in HD patients have been controversial. Some of these studies have indicated positive effects of oral omega-3 supplements on serum systemic inflammatory markers [19-21], while others did not find these effects [22,23]. Furthermore, there is report on positive [19] as well as no effect [24] of oral omega-3 fatty acids on blood hemoglobin level in maintenance HD subjects.

Taking all these data into consideration, our study was aimed to test the hypothesis that administration of omega-3 fatty acids (eicosapentaenoic acid (EPA) and docosahexaenoic acid (DHA)) in maintenance HD patients actually improve systemic inflammation and anemia, and might be useful for reducing serum hepcidin level and consequently required dose of IV iron and EPO.

## Methods

### Subjects

This is a prospective, randomized, single-blind, placebo-controlled trial on patients in two HD centers in Iran (Imam Khomeini Hospital Complex and Sina Hospital affiliated to Tehran University of Medical Sciences, Tehran, Iran). Adult patients who treated with HD for at least 3 months were selected. Patients with pregnancy, malabsorption syndrome, malignancy, inflammatory or infectious diseases, hypothyroidism, medical or surgical illness in recent 3 months, hemoglobinopathies, asthma, chronic obstructive pulmonary disease, coagulopathies, known psychiatric disorders, lack of tolerance or hypersensitivity to fish products as well as those who were receiving corticosteroid, non-steroidal anti-inflammatory drugs, omega-3 fatty acids in the previous three months, anticoagulants including warfarin, immunomodulator or immunosuppressive were excluded.

### Study protocol

The study used a 9-block permuted randomization procedure to allocate subjects randomly into two groups. Each block contained an equal number of omega-3 and control group selections, with the order of the blocks permuted. Random numbers to allocate blocks and randomize group selection were generated using Microsoft Office Excel software. All participants, care providers, and data monitors were blinded to the identity of treatments throughout the study. Patients enrolled into the study were randomized to consume daily dose of either six soft-gel capsules of omega-3 fatty acids (180 mg EPA and 120 mg DHA in each capsule) or matching placebo (containing paraffin oil), provided by Zahravi pharmaceutical company, Tabriz, Iran, for 4 months. Both placebo and omega-3 soft-gel capsules were the same with respect to the size, color, shape, and packaging. All subjects were requested not to alter their habitual diets, drug regimen, and level of physical activity during the study. Adherence to the supplements was assessed by conventional practice of "pill counting", and interviewing with each participant individually thrice weekly. Both omega-3 and placebo capsules were provided free of charge to participants. Furthermore, an attending nephrologist performed a monthly visit for each patient during a routine HD session to encourage adherence to the recommended supplement regimens, and to know adverse effects and tolerability of the supplements. Any changes in the medications as well as patient complaints were recorded during the study period.

At baseline and the end of the study, 10 mL fasting blood were obtained from each patient immediately before HD initiation. The blood samples were centrifuged at 3000 rpm for 10 minutes to separate serum. Serum samples were kept frozen at -70°C for later analysis of serum iron, total iron binding capacity (TIBC), ferritin, hepcidin, iPTH, pro-inflammatory (IL-6, TNF-α, CRP), and anti-inflammatory cytokines (IL-10) concentrations.

The local Ethics Committee of Tehran University of Medical Sciences approved the study protocol, and informed consent was obtained from each patient. The trial was registered in the Iranian Registry of Clinical Trials (registry number: IRCT201202203043N5).

### Measurements

Baseline dialysis adequacy (single pool Kt/V urea) was measured for each subject according to pre- and post-dialysis serum urea nitrogen concentrations, ultrafiltration volume, dialysis time, and post-dialysis weight.

Blood hemoglobin concentration was measured using a cell counter (Sysmex K800, Japan); serum CRP level was measured by a turbidimetric method (Autoanalyzer BT3000, Biotechnica, Italy); serum hepcidin, IL-6, IL-10, and TNF-α were measured using ELISA kits (Bioassay Technology laboratory, Shanghai Crystal Day Biotech Co., Ltd., Shanghai, China).

To evaluate the effect of omega-3 fatty acids on iron status and EPO responsiveness, required dose of IV iron and EPO was recorded monthly over the study course. In addition, EPO resistance index (the ratio of weekly EPO dose to hemoglobin concentration per unit of body weight), as well as TSAT (calculated as serum iron × 100/TIBC) were calculated before and after the 4-month intervention period.

### Statistical methods

Results were presented as means ± SD or medians (interquartile range). The distribution normality of continuous variables was investigated using quantile normal plot, normal probability plot, and Shapiro-Wilk test. Differences between groups were analyzed using unpaired t-test or two-sample Wilcoxon rank-sum (Mann–Whitney) test, while paired t-test or Wilcoxon matched-pairs signed rank test was used to compare differences within groups. The chi-square test was used to compare differences between categorical variables. Moreover, we estimated the effect of omega-3 supplement on hemoglobin, hepcidin, ferritin, TSAT, iPTH and EPO resistance index using the linear regression model with omega-3 supplement as intervention variable. To adjust for the difference in baseline values and also demographic and clinical characteristics, we also entered these variables in the model. Log-transformation was made in case of TSAT before it was included in regression analysis. Additionally, to normalize the amount of IV EPO and iron required to correct anemia, square root of them was created and then were included in regression analysis. Due to non-normality and heterogeneity of variances of the residual distributions, we used non-parametric bootstrap method with 2000 replications to obtain empirical standard errors and bias-corrected and accelerated (BCa) confidence intervals. In addition, because required dose of IV iron and EPO were recorded four times over the course of the study, to estimate the time trend changes in intervention effect, we also fitted a population-averaged panel-data model by using GEE (generalized estimation equation) with autoregressive one correlation structure to account for within-group correlation structure of individuals. P value of less than 0.05 or confidence interval not including null value was considered as statistically significant. All statistical analyses were carried out using the SPSS version 16.0 (SPSS, Inc., Chicago, IL, USA) and Stata software (StataCorp. 2011. Stata Statistical Software: Release 12. College Station, TX: StataCorp LP).

## Results

**Figure 1:**
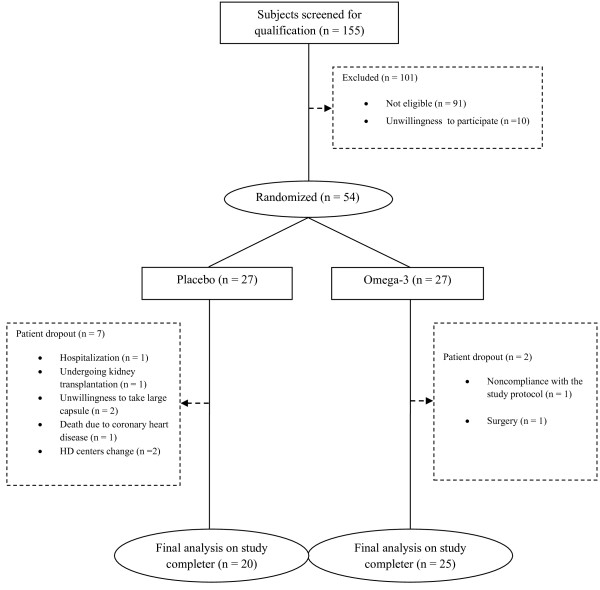
Study’s flowchart indicating patient randomization and participation.

Figure 1 presents patient randomization and participation procedure in the study. Sixty-four patients were qualified for the study. Of those, 54 patients accepted to participate in the trial. Nine subjects dropped out (7 in the placebo and 2 in the omega-3 group) over the course of the study and thus, 45 (20 placebo and 25 omega-3) completed the study, and their data were used in the final analysis. Table 1 summarizes baseline characteristics of subjects who completed the study. Demographic (gender and age) and clinical (HD duration, dialysis adequacy, and underlying causes of ESRD) data of the patients were comparable between the two study groups at baseline.

**Table 1:** Patients’ demographic and clinical data at baseline in the placebo and omega-3 group

**Characteristics**	**Placebo (n = 20)**	**Omega-3 fatty acids (n = 25)**	**P value**
Age (y) (± SD)	57.2 (±15.19)	56.8 (±13.09)	0.925
Sex, n (%)			0.592
Females	8 (40)	12 (48)	
Males	12 (60)	13 (52)	
Hemodialysis duration (mo) (± SD)	72.05 (±60.51)	59.88 (±45.69)	0.450
Dialysis adequacy (Kt/V)	1.27 ± 0.15	1.39 ± 0.22	0.057
Cause of end-stage renal disease, n (%)			0.624
Diabetes	7 (35)	12 (48)	
Hypertension	8 (40)	9 (36)	
Other causes	5 (25)	4 (16)	

Note: Age and hemodialysis duration are expressed as mean ± SD.

Blood hemoglobin levels decreased in both groups during the study period with more decrease in the placebo group. However, the amount of reduction was not significantly different between the two groups (P = 0.316). In addition, although serum iron and TSAT showed an increase in the placebo and decrease in the omega-3 group, the magnitude of changes did not reach statistically significance level between the two groups (Table 2). Mean serum TIBC significantly decreased in the omega-3 group at the end of month 4 compared with the placebo group (P = 0.025). It is worth noting that before within and between group comparisons were performed on serum hepcidin, four major outlying values were excluded (2 from each group) since they were outside the assay range specified in the package insert of hepcidin kit (> 4000 pg/mL). Thereafter, we observed that serum hepcidin level remained unchanged in the placebo group (P = 0.756) at the end of the study compared with baseline, whereas its level increased significantly in the omega-3 group (P = 0.048). However, the mean difference of serum hepcidin were comparable between the two groups (P = 0.145).

**Table 2:** Effects of 4 months omega-3 vs. placebo supplementation on hematological and inflammatory parameters

	**Placebo (n = 20)**				**Omega-3 (n = 25)**				
	**Baseline**	**Month-4**	**Diff**	**P**	**Baseline**	**Month-4**	**Diff**	**P**	**P overall**
**Hgb (g/dL)**									
Mean ± SD	11.14 ± 1.62	10.17 ± 1.62	**-0.97 ± 2**	0.055	11.14 ± 2.27	10.86 ± 1.30	**-0.28 ± 2.21**	0.567	**0.316**
Median (IQR)	10.90 (10.10, 12.30)	10.40 (9.02, 11.20)			11.10 (9.45, 12.15)	10.70 (10, 11.45)			
**Serum iron (μg/dL)**									
Mean ± SD	86.11 ± 37.52	87 ± 43.44	**0.89 ± 49.23**		117.45 ± 117.65	71.85 ± 48.05	**-45.60 ± 115.34**		
Median (IQR)	80 (54.25, 109.50)	66 (53, 132.75)		0.647	66 (48.25, 113.25)	52.50 (45.50, 96)		0.296	**0.781**
**TIBC (μg/dL)**									
Mean ± SD	281 ± 78.09	264 ± 95.29	**-17 ± 125.98**		318.81 ± 75.26	224.60 ± 52.23	**-94.21 ± 77.28**	0.000	**0.025**
Median (IQR)	252.50 (234.50, 297.50)	232 (200, 283.25)		0.446	310 (255, 383)	210 (182, 272)			
**TSAT%**									
Mean ± SD	33.36 ± 16.99	38.56 ± 26.20	**5.20 ± 28.06**		35.15 ± 29.12	32.77 ± 22.19	**-2.37 ± 30.49**		
Median (IQR)	30.41 (19.70, 45.80)	29.19 (18.62, 53.28)		0.711	23.67 (14.08, 42.29)	25.68 (17.65, 42.26)		0.575	**1.000**
**Ferritin (ng/mL)**									
Mean ± SD	345.91 ± 168.90	1236 ± 885.85	**890.05 ± 862.71**		839.63 ± 719.81	1005.10 ± 1185.70	**165.45 ± 886.59**		
Median (IQR)	445.50 (187, 467.50)	1033 (750, 1767)		0.000	696.50 (213.25, 1225.80)	739.50 (126, 1267.50)		0.681	**0.000**
**Hepcidin (pg/mL)**									
Mean ± SD	584.81 ± 654.45	557.25 ± 401.96	**-27.56 ± 650.83**		579.74 ± 340.81	729.48 ± 661.81	**149.74 ± 574.87**		
Median (IQR)	358.50 (306.50, 546.50)	377 (334.50, 812.25)		0.756	392 (318, 983)	443 (335, 856)		0.048	**0.145**
**IL-6 (ng/L)**									
Mean ± SD	103.54 ± 145.18	106.48 ± 136.51	**2.94 ± 206.17**		144.38 ± 167.66	136.85 ± 183.56	**-7.53 ± 126.01**		
Median (IQR)	44.90 (40.22, 87.32)	52.85 (40.55, 98.62)		0.695	56.30 (46.90, 151.45)	50 (29.20, 123.50)		0.034	**0.115**
**TNF-α (ng/L)**									
Mean ± SD	80.93 ± 64.89	77.43 ± 59.82	**-3.50 ± 49.21**		122.09 ± 171.69	116.57 ± 172.32	**-5.52 ± 46.92**		
Median (IQR)	48.05 (42.42, 95.57)	48.05 (44.17, 99.22)		0.616	67 (44.30, 127.20)	58.60 (44.55, 112.80)		0.346	**0.971**
**IL-10 (pg/mL)**									
Mean ± SD	111.76 ± 67.39	107.24 ± 63.01	**-4.51 ± 90.79**		123.94 ± 71.91	132.06 ± 94.81	**8.11 ± 57.63**		
Median (IQR)	78.05 (72.80, 119.08)	83 (68.20, 128.30)		0.446	91.80 (71.80, 154.10)	87.70 (69.90, 149.60)		0.798	**0.445**
**IL-10 / IL-6**									
Mean ± SD	1.57 ± 0.42	1.39 ± 0.35	**-0.18 ± 0.53**		1.32 ± 0.55	1.96 ± 1.19	**0.64 ± 1.14**		
Median (IQR)	1.74 (1.56, 1.79)	1.48 (1.27, 1.66)		0.078	1.48 (0.97, 1.56)	1.59 (1.05, 2.77)		0.013	**0.003**
**IL-10 / TNF-α**									
Mean ± SD	1.85 ± 1.19	1.69 ± 0.95	**-0.16 ± 1.17**		1.35 ± 0.29	1.44 ± 0.28	**0.09 ± 0.25**		**0.399**
Median (IQR)	1.62 (1.26, 2.07)	1.49 (0.91, 2.08)		0.349	1.39 (1.20, 1.58)	1.50 (1.27, 1.64)		0.139	
**CRP (mg/L)**									
Mean ± SD	7.73 ± 4.89	12.69 ± 14.02	**4.96 ± 12.59**		9.24 ± 8.79	8 ± 6.78	**-1.24 ± 5.68**		
Median (IQR)	6.19 (4.79, 8.93)	6.26 (3.55, 18.81)		0.246	6.23 (4.87, 10.26)	5.91 (3.25, 10.72)		0.414	**0.143**
**iPTH (pg/mL)**									
Mean ± SD	271.18 ± 233.60	654.57 ± 1082.30	**383.39 ± 927.49**		333.42 ± 567.22	348.07 ± 723	**14.65 ± 235.88**		
Median (IQR)	180 (95, 507.50)	224 (110.40, 980.50)		0.006	118 (91.35, 316)	167 (97.15, 286)		0.455	**0.17**

Notes: Data are mean ± SD and median (IQR). P values for each group were derived from paired t-test or Wilcoxon signed ranks test between baseline versus month-4 comparison (within group comparison). Overall P values were derived using independent t-test or Mann–Whitney U test, comparing mean differences of parameters between omega-3 and placebo group (between group comparisons of mean differences) (bold faces).

Abbreviations and definitions: IQR, Interquartile range; IL, Interleukin; TNF-α, Tumor necrosis factor alpha; CRP, C-reactive protein; iPTH, Intact parathyroid hormone; Hgb, Hemoglobin; TSAT, Transferrin saturation; TIBC, Total iron binding capacity; Diff, Mean difference of month-4 minus baseline.

Our results demonstrated that omega-3 treated group had less inflammatory state than placebo group at the end of the study, as reflected in the serum concentrations of IL-6, TNF-α, IL-10, CRP, and anti-inflammatory to pro-inflammatory ratios of IL-10 to TNF-α and IL-10 to IL-6. Although serum levels of iPTH and ferritin, as surrogates of systemic inflammation, increased in both study arms during the study, omega-3 treated subjects experienced much less increase than placebo group. Additionally, comparison of mean differences between the two groups became significant with respect to only serum ferritin level and IL-10 to IL-6 ratio (Table 2).

As shown in Table 3, EPO resistance index showed no significant changes in the omega-3 group compared with the placebo group at the end of the study (P = 0.086). At the same time, population-averaged panel-data model did not show any significant change in the required dose of both IV EPO and iron between the two groups in four months of follow-up (Table 4). Of note, omega-3 treatment was weakly and negatively associated with month four IV EPO dose over the course of the study (P = 0.087), whereas no such a finding was found regarding the relation between omega-3 treatment and required IV iron dose (Table 4).

**Table 3:** Mean dose of IV iron and EPO and calculated EPO resistance index in two groups

**Groups**	**Baseline**	**Month 2**	**Month 3**	**Month 4**	**Difference**	**P value**
**IV iron (mg/week)**						
**Omega-3**	65.22 ± 91.93	49 ± 63.11	37 ± 61.29	37.30 ± 76.78	**-27.91 ± 128.96**	**0.554**
**Placebo**	54.17 ± 44.76	36.84 ± 34.73	56.94 ± 71.13	23.61 ± 33.73	**-30.56 ± 64.49**	
**IV EPO (unit/week)**						
**Omega-3**	8695.70 ± 8059	9960 ± 7236.90	8640 ± 7846.80	8956.50 ± 8019.70	**260.87 ± 4013.81**	**0.086**
**Placebo**	6888.90 ± 4861.40	9052.60 ± 4440.60	8333.30 ± 4186.80	9666.70 ± 5005.80	**2777.78 ± 5493.91**	
**EPO resistance index (unit/week per Hgb per weight)**						
**Omega-3**	14.64 ± 15.12	-	-	15.03 ± 13.40	**0.39 ± 7.82**	**0.086**
**Placebo**	11.17 ± 8.72	-	-	17.26 ± 12.02	**6.09 ± 11.89**	

Notes: All values are mean ± SD. Difference indicates mean difference of month-4 minus baseline. P values were derived using independent t-test or Mann–Whitney U test, comparing mean differences of parameters between two groups (bold faces). P value < 0.05 was considered as statistically significant.

*Abbreviations*: *Hgb* Hemoglobin, *IV* Intravenous, *EPO* Erythropoietin.

**Table 4:** Population-averaged panel-data model by using GEE for evaluating the effects of omega-3 supplement on required dose of IV iron and EPO during the study, with time being considered as a possible interacting factor

	**Independent variables**			
**Dependent variable**	**Omega-3†**	**Baseline value**	**Time**	Time*Omega-3
	β = -15.57	β = 0.48	β = 1.71	β = -7.04
**Post-IV EPO dose**	95%CI (-32.93 , 2.93)	95%CI (0.27 , 0.68)	95%CI (-3.24 , 6.36)	95%CI (-17.52 , 2.29)
	P = 0.087	P = 0.000	P = 0.493	P = 0.150
	β = -0.42	β = 0.18	β = -0.73	β = 0.18
**Post-IV iron dose**	95%CI (-3.13 , 2.59)	95%CI (-0.03 , 0.45)	95%CI (-1.94 , 0.66)	95%CI (-1.67 , 2.21)
	P = 0.771	P = 0.166	P = 0.279	P = 0.856

Notes: Square root of intravenous erythropoietin and iron were included in regression analysis.

*Abbreviations*: *EPO* Erythropoietin, *IV* Intravenous, *Post*- Post-intervention, *TSAT* Transferrin saturation, *CI* Bias-corrected confidence interval.

**†**The coefficient indicates predictive value of omega-3 treatment on the difference in square root of outcome variables measured at the end of the study between omega-3 and placebo groups.

P value < 0.05 or 95%CI not including null value was considered as statistically significant.

Regression analysis with bootstrap method illustrated that among anemia parameters studied in this study, omega-3 treatment was a significant predictor of only serum ferritin level after adjustment for patients’ baseline demographic and clinical characteristics (Table 5).

**Table 5:** Effects of omega-3 supplement on anemia parameters and iPTH in multiple linear regression model

		**Dependent variable†**					
**Independent variable**		**Post-Hemoglobin**	**Post-Hepcidin**	**Post-Ferritin**	**Post-TSAT**^**ψ**^	**Post-EPO resistance index**	**Post-iPTH**
**Omega-3 supplement**	Coefficient (β)	0.683	67.979	-922.484	-0.273	-5.404	-414.247
	Bootstrap standard error	0.547	147.169	282.992	0.218	3.899	213.935
	P value	0.212	0.644	0.001*	0.211	0.166	0.053
	95% CI	(-0.361, 1.776)	(-203.593, 362.789)	(-1533.274, -412.422)	(-0.671, 0.193)	(-12.844, 2.466)	(-**1150.008, -129.641**)*

*Abbreviations*: *EPO* Erythropoietin, *Post*- Post-intervention, *TSAT* Transferrin saturation, *iPTH* Intact parathyroid hormone, *CI* Bias-corrected and accelerated confidence interval.

**†**All analyses were adjusted for age, gender, hemodialysis duration, and baseline values of Kt/V and outcome variables.

**Ψ**Both baseline and post-intervention TSAT were included in regression analysis in logarithmic scale.

*P value < 0.05 or 95%CI (with bootstrap method) not including null value was considered as statistically significant.

## Discussion

Anemia occurs as a common complication in patients with chronic kidney disease and associated with a poor quality of life and bothersome symptoms including fatigue, headache, dyspnea, and light-headedness [25]. Notably, it contributes to the high prevalence of left ventricular hypertrophy in chronic kidney disease patients, even prior to the onset of dialysis, which has been associated with cardiovascular mortality [26]. Thus, careful detection and treatment of anemia is of clinical importance in dialysis patients.

We aimed to investigate the potential benefits of omega-3 fatty acids as adjunct to IV iron and EPO treatment in correction of anemia in maintenance HD patients. Our study showed that administration of omega-3 fatty acids for 4 months caused no change in blood hemoglobin level. This finding was in line with that of Kooshki *et al*.’s study, in that they showed no significant change in blood hemoglobin level following a 10-week supplementation of HD patients with 2080 mg omega-3 fatty acids per day [24]. Additionally, Donnelly *et al.* illustrated that daily ingestion of 3.6 g omega-3 fatty acids by HD patients for 4 weeks produced no significant change in blood hemoglobin concentration [27]. Conversely, Perunicic-Pekovic *et al.* indicated that daily supplementation of HD patients with 2.4 g omega-3 fatty acids for 8 weeks caused a significant increase in blood hemoglobin concentration [19]. The observed discrepancies may in part be related to the diversity in study design, duration and dosage of omega-3 supplementation, varied dietary intake, and baseline blood hemoglobin concentration of the patients.

Supplemental use of omega-3 fatty acids for 4 months also failed to increase TSAT as representative of available serum iron for erythropoiesis. Although we found no similar study in available literature regarding the effect of omega-3 fatty acids on available serum iron to be compared with our results, it seems that inability of omega-3 supplement to improve TSAT might be related to lower dosage of omega-3 supplement (1800 mg per day), relatively favorable TSAT at baseline, or uncontrolled dietary intake of iron.

Moreover, serum TIBC showed a significant reduction in the omega-3 group compared to the placebo group over the course of the study. This finding was in contrast to that of Kalantar-Zadeh *et al*.’s study, in which they observed a significant increase in serum TIBC in hypoalbuminemic HD patients who consumed a nutritional oral supplement containing 1680 mg omega-3 fatty acids [28]. Although the supplement was used during each HD session for 4 weeks, the complex composition of the supplement makes it impossible to distinguish the potential positive effect of omega-3 fatty acids on serum TIBC from other constituents. In addition, Szklarek-Kubicka *et al.* found no marked effects of intradialytic intravenous administration of omega-3 emulsion (containing 2g EPA and 2g DHA) on serum transferrin after 11 consecutive HD sessions [29]. Because dietary protein intake can influence serum transferrin concentration, as a correlate of TIBC, uncontrolled patients’ diet in our study might have led to this controversial result. Altogether, considering no significant change in TSAT along with relative attenuated inflammatory state in the omega-3 group, conflicting result of our study remains to be elucidated in more controlled studies.

In the present work, serum ferritin level increased in both groups during the study period with much more retarded increase in the omega-3 group. In contrast, Rasic-Milutinovic *et al.* showed that daily administration of 2.4 g omega-3 fatty acids for 8 weeks significantly lowered serum ferritin level in maintenance HD patients [21]. Huang *et al.* failed to demonstrate an association between plasma omega-3 fatty acids concentration and systemic inflammation in dialysis patients [30]. We used a moderate dose of 1800 mg omega-3 supplement per day for 4 months, which may be insufficient to replace membrane phospholipids’ fatty acids beside plasma free fatty acids to exert prominent anti-inflammatory effects. However, omega-3 supplement in our study was a significant and independent predictor of reduction in serum ferritin. Given the unchanged TSAT in both arms of this study, it is conceivable that changes in serum ferritin, as an acute phase protein, was more reflective of systemic inflammation rather than iron stores. This concept was further supported when serum ferritin increased more restrictedly in omega-3 treated patients who experienced slight alleviation in systemic inflammation (Table 2).

The mean difference of required IV EPO dose in the omega-3 group was lower than that in the placebo group (Table 3). Consistently, population-averaged panel-data model revealed that omega-3 supplement was a weakly predictor of reduced IV EPO dose in month four of intervention. Therefore, more pronounced reduction in IV EPO dose might likely appear with omega-3 supplementation if the study period was expanded. In contrast, Kooshki *et al.* found no significant changes in mean dose of IV EPO in either omega-3 (2080 mg/day) or placebo group during their 10-week study period [24]. In our study, EPO resistance index indicated no significant improvement in omega-3 treated subjects compared with the placebo group during the study period. In contrast to this finding, Himmelfarb *et al.* demonstrated that daily administration of gamma-tocopherol-enriched mixture of DHA (containing 924 mg gamma-tocopherol and 1200 mg DHA) for 8 weeks caused a significant reduction in the serum IL-6 and erythropoietic index (weekly EPO dose divided by hematocrit) in maintenance HD patients [31]. In spite of the contributory role of inflammation in the EPO resistance [32-34], relative attenuated inflammation by omega-3 supplement in our study was not sufficient to cause favorable changes in EPO resistance.

Unexpectedly, required dose of IV iron decreased in both groups through the study period with no significant difference between the mean differences of the two groups (Table 3). Additionally, population-averaged panel-data model showed no role of omega-3 supplement over time in the prediction of post-treatment IV iron dose. Unfortunately, we were not able to interpret such a finding using available data.

In our study, although omega-3 treated patients experienced significant increase in the ratio of IL-10 to IL-6 and marked favorable changes in the serum ferritin level compared to the placebo group, other serum inflammatory markers including IL-6, TNF-α, CRP, IL-10, iPTH and IL-10-to-TNF-α ratio also desirably changed in this group. Inability of omega-3 supplement to provide marked anti-inflammatory effects in this study may be due to limited statistical power, small dosage of omega-3 fatty acids supplement, shorter observation period, or all of these. It should be mentioned that results of the majority of the studies evaluating the effects of omega-3 fatty acids on inflammatory markers in HD patients are inconclusive, largely due to diversity in study design, supplement dosage, and study length. Saifullah *et al.* showed that daily administration of 1.3 g omega-3 fatty acids for 12 weeks induced a significant reduction in serum CRP levels of HD patients [20]. In addition, Perunicic-Pekovic *et al.* demonstrated significant decline in serum IL-6 and TNF-α levels of HD subjects following daily consumption of 2.4 g of omega-3 fatty acids for 2 months [19]. Bowden *et al.* treated maintenance HD patients with 1560 mg omega-3 fatty acids per day for 6 months, and found a significant decrease in serum CRP level [35]. In contrast, a recent study by Kooshki *et al.* failed to demonstrate any effects of omega-3 fatty acids (2080 mg per day for 10 weeks) on serum CRP, TNF-α, and IL-6 concentration in HD patients [36]. In agreement with our results, Hassan *et al.* found insignificant decrease in the serum concentrations of CRP, TNF-α, and IL-6 after 8 weeks of oral omega-3 supplementation (3.4 g EPA and DHA per day) in peritoneal dialysis patients [37]. Also our results were in line with those of Poulia *et al*.’s study, that daily ingestion of 1680 mg omega-3 fatty acids for 4 weeks caused an insignificant decline in serum CRP concentrations in chronic HD subjects [38].

Additionally, omega-3 supplement was a significant predictor of reduced serum iPTH level in our study (Table 5). None of the participants ingested cinacalcet to control hyperparathyroidism, and the number of subjects receiving calcitriol was comparable between the two study arms. Thus, significant association of omega-3 supplementation with reduced serum iPTH levels might largely be related to partial decline in systemic inflammation following 4 months of omega-3 administration.

In addition, serum hepcidin level showed no significant change in the omega-3 group compared with the placebo group. There are conflicting reports with respect to the relation between inflammation markers and serum hepcidin. Some studies have detected a positive association [39,40], while others have not [15,41,42]. Our study showed a relative improvement in systemic inflammation by omega-3 treatment, whereas serum hepcidin level showed a significant increase in this group. Also TSAT, as an important regulator of hepcidin synthesis [43], had no significant changes within and between the two groups during the study period. Actually, we found no similar study in available literature regarding the effect of omega-3 supplement on serum hepcidin to compare our result. However, some reasons could be provided for the observed discrepancy. First, dosage of omega-3 fatty acids used in our study might not be sufficient to exert pronounced suppression on systemic inflammatory markers in general, and serum hepcidin in particular. Second, considering normal value of inflammation markers in healthy individuals [44,45], our study subjects had no overt sever inflammation at baseline or at the end of the study, that this state might partly weaken the anti-inflammatory effect of omega-3 fatty acids on hepcidin production. Third, elevated serum hepcidin may not be necessarily needed to maintain a mild iron deficiency and anemia of inflammation because other inflammatory mediators have a similar effect, and normal serum hepcidin level is also able to sufficiently restrict iron supply and prevent the correction of the established anemia [41]. Finally, interrelations among the response to EPO, serum iron status, iron therapy, and serum hepcidin increase the complexity of interpretations in this area. For instance, some iron parameters are representative of both iron stores and inflammation (i.e. ferritin) or have a mixed inflammatory and nutritional significance (i.e. transferrin) [46,47].

Our study may be criticized for some limitations, as follows: low statistical power to detect meaningful differences between the groups due to the small sample size and short observation period, monitoring of patient compliance with "pill counting" practice instead of in vivo measurement of omega-3 fatty acids, lack of interim analysis on all outcome variables to identify a potential trend over time and reduce variance of estimates of treatment effects, distortion of subject blindness in the placebo arm due to "Fishy" smells of omega-3 soft-gel capsules, and lack of careful assessment of patients’ dietary intake. Despite the above-mentioned limitations, our trial had several strengths including placebo-controlled design, relatively longer duration of the study compared with previous ones, relatively comprehensive assessment of anemia and inflammation markers, absent serious adverse effects causing patient withdrawal, and enhanced patient adherence via more frequent interviews compared with previous studies.

In summary, the present study showed a mild attenuation in systemic inflammation of chronic HD patients by omega-3 fatty acids supplement. Although omega-3 supplement failed to improve anemia, its positive, though slight, effects on serum inflammation markers including ferritin were encouraging. Considering elevated serum hepcidin in the presence of unchanged TSAT and weakened inflammation in the omega-3 group, it can be speculated that other impressive factor(s) not controlled in our study might contribute to these contradictory results. Therefore, there is a need for further well-designed studies to precisely determine utility of omega-3 fatty acids in HD patients with respect to inflammation and anemia.

## Competing interests

The authors declare that they have no competing interest.

## Authors’ contribution

AG: made substantial contributions to study design, acquisition and interpretation of data and drafting the manuscript and revising it. MRK: have been involved in drafting the manuscript and revising it critically for important intellectual content. SDK: have made substantial contributions to conception and design of the study, have been involved in drafting the manuscript or revising it critically for important intellectual content, and final approval of the manuscript. ER: made substantial contributions to data acquisition. AA: made substantial contributions to data acquisition. SSHN: made substantial contributions to data analysis. MAM: made substantial contributions to data analysis. All authors read and approved the final manuscript.
